# Temperature and Pressure Effects of Desalination Using a MFI-Type Zeolite Membrane

**DOI:** 10.3390/membranes3030155

**Published:** 2013-07-17

**Authors:** Bo Zhu, Jun Hyun Kim, Yong-Han Na, Il-Shik Moon, Greg Connor, Shuichi Maeda, Gayle Morris, Stephen Gray, Mikel Duke

**Affiliations:** 1 Institute for Sustainability and Innovation, College of Engineering and Science, Victoria University, Hoppers Lane, Werribee 3030, Australia; E-Mails: bo.zhu@vu.edu.au (B.Z.); stephen.gray@vu.edu.au (S.G.); 2 Chosun Refractory Co. Ltd., Taein Dong, Kwangyang-si, Jeonlanam-do 545-893, Korea; E-Mails: jhkim82@chosunref.co.kr (J.H.K.); yongna@chosunref.co.kr (Y.-H.N.); 3 Department of Chemical Engineering, Sunchon National University, Maegok Dong, Suncheon 540-742, Korea; E-Mail: ismoon@sunchon.ac.kr; 4 C.I. Ceramics (Aust.) Pty. Ltd., Rivulet Crescent, Albion Park Rail 2527, Australia; E-Mails: greg.connor@ciceramics.com.au (G.C.); shuichi.maeda@ciceramics.com.au (S.M.); 5 Research Services Office, Flinders University, Adelaide 5001, Australia; E-Mail: gayle.morris@flinders.edu.au

**Keywords:** desalination, MFI-type zeolite membrane, rubbing method, seeded secondary growth

## Abstract

Zeolites are potentially a robust desalination alternative, as they are chemically stable and possess the essential properties needed to reject ions. Zeolite membranes could desalinate “challenging” waters, such as saline secondary effluent, without any substantial pre-treatment, due to the robust mechanical properties of ceramic membranes. A novel MFI-type zeolite membrane was developed on a tubular α-Al_2_O_3_ substrate by a combined rubbing and secondary hydrothermal growth method. The prepared membrane was characterised by scanning electron microscopy (SEM), X-ray photoelectron spectroscopy (XPS) and single gas (He or N_2_) permeation and underwent desalination tests with NaCl solutions under different pressures (0.7 MPa and 7 MPa). The results showed that higher pressure resulted in higher Na^+^ rejection and permeate flux. The zeolite membrane achieved a good rejection of Na^+^ (~82%) for a NaCl feed solution with a TDS (total dissolved solids) of 3000 mg·L^−1^ at an applied pressure of 7 MPa and 21 °C. To explore the opportunity for high salinity and high temperature desalination, this membrane was also tested with high concentration NaCl solutions (up to TDS 90,000 mg·L^−1^) and at 90 °C. This is the first known work at such high salinities of NaCl. It was found that increasing the salinity of the feed solution decreased both Na^+^ rejection and flux. An increase in testing temperature resulted in an increase in permeate flux, but a decrease in ion rejection.

## 1. Introduction

Membranes are now the state-of-the-art for water treatment, including most new desalination plants. However, the reverse osmosis (RO) membranes used for desalination are polymeric-based and have some common problems, such as biofouling, oxidation, metal oxide fouling, abrasion and clay and mineral scaling [1]. Polymeric RO membranes require strict pre-treatment, such as particle removal and removal of oxidants, such as chlorine. Despite the strict pre-treatment requirements, they eventually need to be replaced around every 5–7 years, due to damage caused either by foulants or the chemicals used for cleaning [2]. Therefore, research of alternative materials, such as inorganic membranes for desalination, is needed to address these material-based limitations. 

Nanoporous inorganic membranes have been studied both theoretically and experimentally to reject ions by filtration, utilising single layers [3] and a novel bilayer concept [4,5]. There have been some studies to date applying different membrane materials, such as zeolites [3,6,7,8,9,10,11], and hybrid organically bridged silica [5] for separation of salt from aqueous solutions. Zeolite materials are highly configurable through their chemistry and offer unique frameworks for a wide variety of applications, including chemical sensing, water treatment and chemical reaction [3,6,7,8,9,10,11,12,13,14]. Zeolites are crystalline, hydrated aluminosilicates [15], naturally formed or synthesised, with open structures, which may incorporate a range of small inorganic and organic species. The frameworks of the zeolitic materials, which form the channels and cavities, are constructed from tetrahedral groups (e.g., AlO_4_, SiO_4_, PO_4_, BeO_4_, GaO_4_, GeO_4_ and ZnO_4_) linked to each other by sharing of oxygen atoms [16]. The most common zeolites are based on AlO_4_ and SiO_4_ tetrahedrals linked together to form a three-dimensional network having pores of comparable molecular dimensions to many chemical substances [16,17,18]. The remarkable porous crystalline aluminosilicate structure of zeolites has led to their wide application as molecular sieves for the separation of gases and liquids [19]. 

Ceramic membranes made from zeolites have been shown to be promising candidates for desalination of saline water, including seawater, as they possess the nanoporous structure required to reject ions [6,11]. Zeolite membranes offer a chemically robust desalination option to desalinate “challenging” waters or even reduce the cost of current desalination by reducing the pre-treatment, replacement and cleaning costs of current polymer membrane technology. Zeolite membranes may be used as an alternative to polymeric membranes for treatment of complex wastewater containing organic solvents and radioactive elements [6,10]. Since a molecular dynamic simulation study carried out by Lin and Murad [11] demonstrated that zeolite pore structure is ideally suited to reject ions, several research groups have explored the possibility of using MFI-type zeolite membranes for desalination [6,7,8,9,10]. The MFI-type zeolite has orthorhombic crystal symmetry with nearly cylindrical, 10-member ring channels [20]. The aperture size of the MFI-type zeolite is approximately 0.56 nm [8], which is smaller than the sizes of hydrated ions (e.g., Na^+^ 0.716 nm, Cl^−^ 0.664 nm), but larger than the kinetic diameter of water (0.276 nm) [21]. Performance testing of MFI type zeolite membranes working in reverse osmosis demonstrated that high rejections of even the smallest ions, including Na^+^, are achievable [6,9]. Recently, researchers have also attempted to treat oily water, produced water and radioactive solutions using zeolite membranes [22,23,24,25]. It was found that zeolitic membranes had great potential for separation of dissolved organics from aqueous solution and can also be used to treat low level radioactive wastes through the pervaporation process. However, little work has been carried out to explore the influence of high salinity and temperatures on the desalination performance of zeolite membranes. 

In this study, we deposited MFI-type zeolite seeds on the outer surface of a tubular α-Al_2_O_3_ support using the rubbing method [26,27] and then used secondary hydrothermal growth to prepare a MFI-type zeolite membrane. The prepared membrane was evaluated by gas permeation (He and N_2_) and then underwent desalination performance testing with NaCl solutions under different conditions. The effects of NaCl feed concentration, applied pressure and temperature on membrane performance were investigated. 

## 2. Results and Discussion

### 2.1. Gas Permeation

Gas permeation was used to evaluate the intactness of the zeolite membrane. The permeation of single gas (He or N_2_) measured for the prepared zeolite membrane is shown in Table 1. The gas permeation results for the bare α-Al_2_O_3_ support are also included in Table 1 for comparison. Since the membrane could adsorb water from air and water molecules can occupy the tight micropores of the zeolite structure and, thus, affect the gas permeability of the membrane, a gas permeation test was carried out on the zeolite membrane before and after drying at 100 °C in air to eliminate this effect. It can be seen from Table 1 that the permeance of He or N_2_ for the membrane measured was ~30–40-times smaller than that of the bare tube, indicating that a rate limiting zeolite layer was formed on the surface of the support. The He/N_2_ permselectivities (determined by the ratio of single gas permeances) for the membrane were smaller than 2.6 (Knudsen He/N_2_ permselectivity), suggesting that the membrane might have pores larger than those capable of Knudsen selectivity. However, there is no evidence to show that salt rejection correlates with He/N_2_ permselectivity. 

**Table 1:** Permeation of He and N_2_ for the bare α-Al_2_O_3_ support and zeolite membrane before and after drying at 100 °C in air for 1 h. For reference, the Knudsen permselectivity of He to N_2_ is 2.6.

Sample	He permeation (mol·m^−2^·s^−1^·Pa^−1^)	N_2_ permeation (mol·m^−2^·s^−1^·Pa^−1^)	He/N_2_ permselectivity
Bare tube—new	39.1 × 10^−7^	27.6 × 10^−7^	1.4
Bare tube—dry	30.5 × 10^−7^	24.6 × 10^−7^	1.2
Membrane—new	1.3 × 10^−7^	0.7 × 10^−7^	1.8
Membrane—dry	1.3 × 10^−7^	0.7 × 10^−7^	1.8

### 2.2. Desalination Performance

To explore the opportunity for high salinity and high temperature desalination, the performance of the prepared MFI-type zeolite membrane was evaluated by NaCl solutions with different concentrations (e.g., NaCl concentrations of 3000 to 90,000 mg·L^−1^) under various operating conditions (e.g., pressure of 0.7 MPa or 7 MPa and temperature of 21 °C or 90 °C). The high salt concentrations (>35,000 mg·L^−1^) tested in the current work reflect the typical upper limit of commercial polymeric RO membranes used to desalinate seawater. However, polymer desalination membranes generally cannot be operated at 90 °C, and the exploration in this work presents a novel opportunity for high temperature desalination. An application of high temperature desalination is in cases of naturally hot water (e.g., hot saline groundwater) and saline waters in industry that would normally be cooled to <40 °C to allow treatment by polymer RO membrane. The osmotic pressures needed for desalination of the above NaCl solutions were estimated by Equation (1) [28] and are summarised in Table 2. The effective pressure for NaCl solutions with different concentrations can also be calculated according to Equation (2):

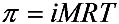
(1)

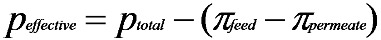
(2)
where π is the osmotic pressure (atm), *i* is the dimensionless Van ’t Hoff factor, *M* is the molarity of the salt in solution, *R* is the ideal gas constant (0.0821 L·atm·K^−1^·mol^−1^) and *T* is temperature (K). For simple binary electrolytes, like NaCl, *i* = 1.8 represented incomplete dissociation (ion pairing) [28]. *p_effective_* is the effective pressure (MPa), *p_total_* is the applied gauge pressure (MPa) and *π_feed_* and *π_permeate_* are the osmotic pressures (MPa) calculated from Equation (1) for the feed solution and permeate, respectively.

**Table 2:** Osmotic pressures determined by Equation (1) for the conditions used in this work.

NaCl concentration (mg·L^−1^)	Temperature (°C)	π (MPa)
3000	21	0.23
3000	90	0.28
35,000	21	2.6
50,000	21	3.8
70,000	21	5.3
90,000	21	6.8

**Figure 1:**
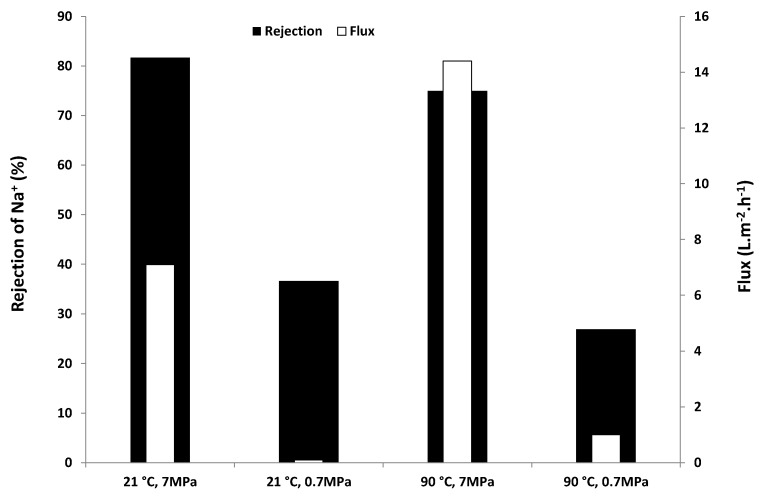
Na^+^ rejection and flux on the zeolite membrane for the NaCl feed solution (total dissolved solids (TDS) of 3000 mg·L^−1^) under different applied pressures and temperatures.

The results for desalination of a NaCl solution with low salt concentration (TDS 3000 mg·L^−1^) under different pressures and temperatures are shown in Figure 1. It can be seen that the prepared MFI-type zeolite membrane achieved a reasonable Na^+^ rejection (~82%) with a flux of ~7 L·m^−2^·h^−1^ for a 3000 mg·L^−1^ NaCl feed solution at a high applied pressure of 7 MPa and 21 °C. Further testing with the same feed solution under a low applied pressure of 0.7 MPa and at the same temperature (21 °C) showed a lower level of Na^+^ rejection (~37%) and a significant decline in flux (~0.1 L m^−2^·h^−1^) when compared with the results obtained at 7 MPa. This result is supported by previous research [8] where rejection increased with pressure on zeolite membranes, because water is more strongly affected by total pressure. Li and co-workers [6] also reported a Na^+^ rejection of ~77% on an MFI-type zeolite membrane with a stabilised water flux of 0.112 L·m^−2^·h^−1^ for a single 0.1 M NaCl (5850 mg·L^−1^) feed solution with a transmembrane pressure of 2.07 MPa. While the Na^+^ rejection (~37%) achieved in this study for a single NaCl feed solution of 3000 mg·L^−1^ under an applied pressure of 0.7 MPa is practically too low, the current work aims to explore the effects of high salinity, pressure and temperature on membrane performance.

The results obtained from the tests at 90 °C at different pressures showed the same trend for Na^+^ rejection and flux as that of 21 °C. However, a further decrease in the rejection of Na^+^ and a significant increase in the flux for both applied pressures (0.7 MPa and 7 MPa) were observed when increasing the testing temperature from 21 °C to 90 °C (Figure 1). 

These results appear to follow the effect related to change in effective pressure [Equation (2)]. The osmotic pressures of the solutions used in this work are shown in Table 2. Based on the finding that water is most sensitive to pressure, decreasing rejection is clear when pressure is reduced, as less effective pressure is available for RO. For example, the effective pressure [estimated from Equation (2)] decreased from 6.8 MPa to 0.6 MPa when the applied gauge pressure was reduced from 7 MPa to 0.7 MPa. Furthermore, there is a slight increase in osmotic pressure with increased temperature, which would also lead to a reduced rejection, especially at low applied pressures (e.g., 0.7 MPa). Normally, this would happen in tandem with reduced flux, but activated diffusion was accelerated beyond this effect. An increase in permeate flux with an increase in temperature was also observed by Li *et al.* [8]. They found in their study that increasing the feed temperature up to 50 °C significantly increased both water and ion fluxes. Possible changes in the pore characteristics of the membrane’s MFI structure [29] and a decrease in the extent of hydration of ions [30] when increasing the temperature may also contribute to the increase in flux and decrease in ion rejection. A dedicated diffusion study is required to properly investigate the diffusion of water and ions coupled with the effects of osmotic pressure and temperature-driven diffusion of both ions and water molecules.

**Figure 2:**
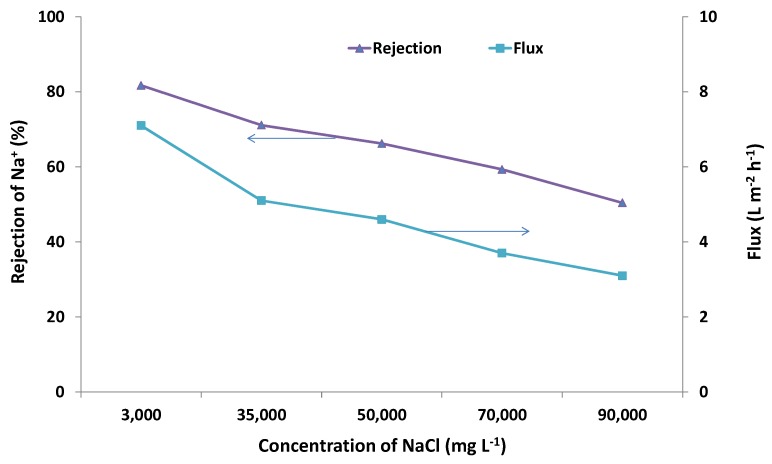
Na^+^ rejection and flux on the zeolite membrane for the feed solutions with different NaCl concentrations (TDS 3000 to 90,000 mg·L^−1^) at an applied gauge pressure of 7 MPa and 21 °C.

**Figure 3:**
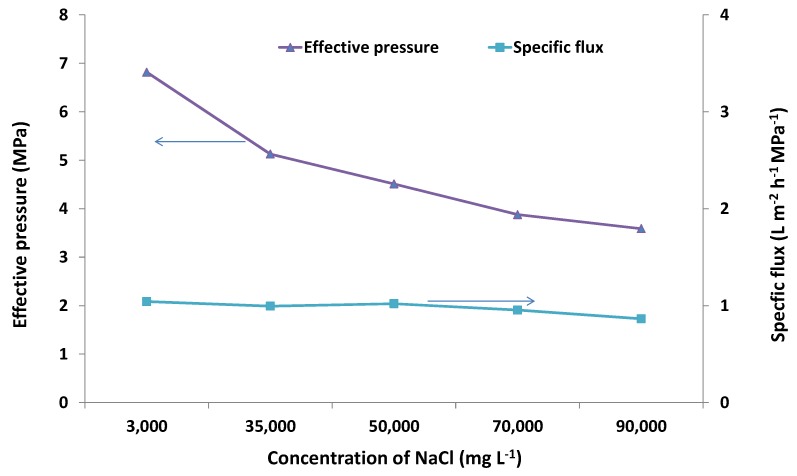
Effective pressure and specific flux on the zeolite membrane for the feed solutions with different NaCl concentrations (TDS 3000 to 90,000 mg·L^−1^) at an applied pressure of 7 MPa (gauge) and 21 °C.

In the current work, the effect of the solution salinity on membrane performance was also investigated. Figure 2 compares the results from further desalination tests on different concentrations of NaCl feed solutions (35,000, 50,000, 70,000 and 90,000 mg·L^−1^) at 7 MPa and 21 °C with those obtained for 3000 mg·L^−1^ at the same conditions. Figure 3 shows the effect of salinity on the effective pressure [estimated from Equation (2)] and specific flux, which was estimated according to the following equation:

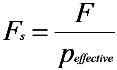
(3)
where *F_s_* (L·m^−2^·h^−1^·MPa^−1^) is the specific flux and *F* (L·m^−2^·h^−1^) is the flux obtained from Figure 2 for a certain tested NaCl concentration.

The results showed that both Na^+^ rejection and flux decreased with increasing salinity (Figure 2). Rejection decreased from around 80% to 50% when the concentration of the feed NaCl solution was increased from 3000 to 90,000 mg·L^−1^. Flux decreased from 7 to 3 L·m^−2^·h^−1^ over the same concentration range tested. This is reasonable, as a higher salinity solution needs higher osmotic pressure (Table 2), thus resulting in less effective pressure available for RO compared to that of a lower salinity solution under the same applied pressure (Figure 3). However, regardless of the higher concentration tested in this study, rejection decreased, such that the osmotic pressure difference remained below the applied pressure (7 MPa). For example, at 90,000 mg·L^−1^, rejection decreased to 50%, so this salinity difference was 45,000 mg·L^−1^, corresponding to an osmotic pressure of about 3.4 MPa [based on Equation (1)], which is around half of the applied pressure (Figure 3). It can also be seen from Figure 3 that the specific flux remained almost unchanged when the concentration of the feed NaCl solution was increased from 3000 to 90,000 mg·L^−1^. This confirmed that the decrease in flux with the increased salinity of feed NaCl solutions was caused by changes in the effective driving pressure.

### 2.3. SEM

**Figure 4:**
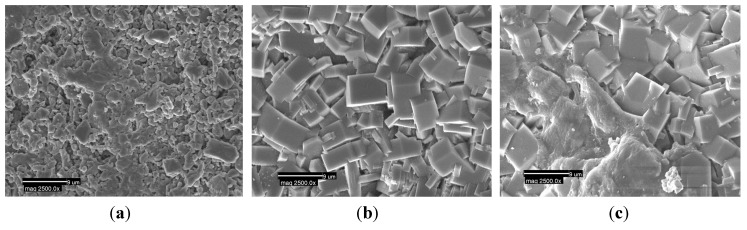
SEM images on the surface of (**a**) α-Al_2_O_3_ substrate; (**b**) original zeolite membrane; (**c**) desalination tested (~90 h) zeolite membrane.

SEM was employed to investigate the morphology of the zeolite membrane before and after ~90 h desalination testing with NaCl solutions. Figure 4 shows the SEM images of the surface of the bare α-Al_2_O_3_ support and the top layer of the MFI-type zeolite membrane. The uncoated support presented plate-like particles with high porosity (Figure 4a). The image (Figure 4b) of the as-synthesised zeolite membrane surface showed typical rarndomly orientated MFI-type zeolite crystals, confirming the formation of a zeolite layer on the α-Al_2_O_3_ support, as determined by gas permeation (Section 2.1). Most of the zeolite crystals laid randomly on the surface of the α-Al_2_O_3_ support. Although SEM was not carried out on the cross-section of the membrane in this study, we expect the membrane thickness to be ~3 µm, as measured for the other membranes prepared in our laboratory by the same procedures as used for the current work. The top view (Figure 4c) of the surface of the tested membrane showed no significant change to the membrane structure after ~90 h desalination testing under different conditions (e.g., pressures, temperatures). The membrane retained only some “loose” deposition of salts on the surface after ~90 h desalination testing with NaCl solutions. The recent stability study carried out by Drobek, *et al.* [31] on zeolite membranes showed some permanent decay after up to 560 h desalination testing in pervaporation mode. This was due to the combined effects of ion exchange and water dissolution mechanisms. Despite this finding, we did not see a membrane performance loss in our study during a relatively shorter term (~90 h) of desalination testing with NaCl solutions in filtration mode.

### 2.4. XPS

Elemental analysis was also conducted by XPS on the surface of the original and desalination tested MFI-type zeolite membrane to determine elemental changes after salt exposure. Prior to XPS measurements, the desalination tested membrane was permeated with deionised water and rinsed to remove loosely bound material (including ions). As shown in Table 3, Na and Cl signals were detected on the surface of the desalination tested membrane by XPS elemental analysis. This suggests that zeolites interacted with ions present in NaCl feed solution during desalination. The interactions between the MFI-type zeolites and ions when exposed to seawater or a single solution (NaCl or KCl) were also observed in our previous material adsorption studies [32,33]. Cl was also detected in the material, which has not been analysed in our previous studies [32,33]. Cl surface concentration was less than the Na surface concentration after desalination testing, potentially indicating a preference for Na in the negatively charged material [9]. It is clear from this result that XPS observes the presence of Na more strongly than Cl. The X-rays will penetrate quite significantly into the sample, several microns at least, but excited electrons leave with very low energy and, thus, can only escape from the top 1–10 nm of the surface (the typically analysis is at a depth of 5 nm) [34,35,36]. Therefore, Cl must be residing in locations shielded from the XPS analysis, potentially deeper within the grain boundaries, as opposed to the MFI zeolite surface and intrinsic pores.

**Table 3:** XPS analysis of elements on the bare α-Al_2_O_3_ substrate, the original and the desalination tested zeolite membrane.

Element	Bare tube (at %)	Original membrane (at %)	NaCl tested membrane (at %)
O 1s	18.3	31.2	35.4
C 1s	54.8	40.8	33.2
Al 2p	15.9	2.0	–
Si 2p	3.0	22.7	24.7
Ca 2p	1.6	1.3	0.9
N 1s	2.4	2.0	2.7
Cl 2p	0.8	–	0.9
Na 2s	3.2	–	2.2

## 3. Experimental Section

### 3.1. Preparation of MFI-Type Zeolite Membrane

**Figure 5:**
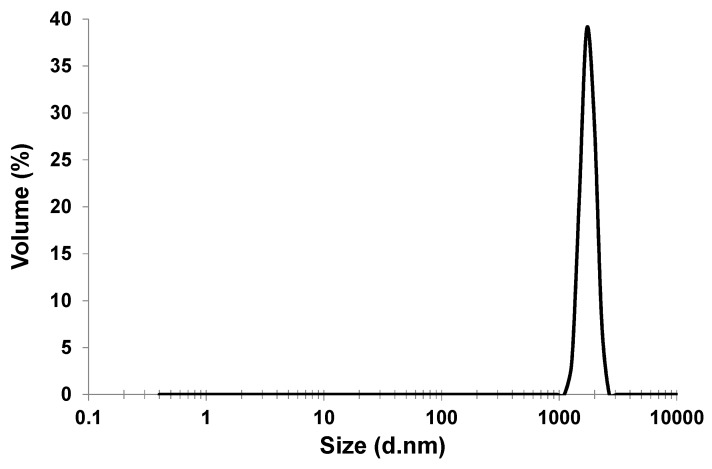
Zetasizer measured particle size distribution of the zeolite seeds.

The MFI-type zeolite membrane was coated on a porous α-Al_2_O_3_ tubular support (external diameter 15 mm, internal diameter 10 mm, length 25 mm, ~0.58 µm nominal pore size, Chosun Refractory Co. Ltd., Korea) by a secondary growth technique, which involved depositing zeolite seeds on the support using a rubbing method [26,27] followed by growth of the membrane under hydrothermal conditions [9]. Prior to membrane preparation, the bare α-Al_2_O_3_ tube was tested under high pressure up to 10 MPa, confirming that the ceramic tube used in this study for membrane preparation can withstand the desalination test system desired pressure (e.g., 7 MPa). The zeolite seed powders (ZSM-5, SiO_2_/Al_2_O_3_ = 360) used for seed-deposition were supplied by ACS Material, USA. The particle size distribution of the zeolite seeds used in this study was determined by a Zetasizer (Malvern Instruments-nano-series) to be between 1000 nm and 3000 nm (peaking at ~1800 nm) in our laboratory (Figure 5). The hydrothermal secondary growth was carried out in a growth solution of 2 mL of 1M tetra-propyl ammonium hydroxide (TPAOH) (Aldrich), 2 mL of tetraethyl orthosilicate (TEOS) (98%, Aldrich) and 36 mL DI water at 180 °C for 16 h. After growth, the membrane was washed in deionised water to remove loose precipitate and was then calcined at 500 °C for 4 h. 

### 3.2. Characterisation

The original and desalination tested MFI-type zeolite membrane was characterised by SEM and XPS to determine any changes in membrane structure, morphology and surface elements after long-term desalination testing under different conditions. Prior to SEM and XPS measurements, the desalination tested membrane underwent deionised water permeation to remove adsorbed material (including ions). 

SEM images were obtained from the secondary electron detector of a CamScan MX2500 microscope (CamScan Optics, Cambridge, UK) using a 10 kV electron beam with a working distance of 2.2 mm.

XPS spectra were recorded using a Leybold-Heraeus “LHS-10” XPS instrument, with EA-10/100 concentric hemispherical analyser, operating in constant retarding ratio mode for survey scans and constant analysis energy mode for high resolution scans. The base pressure was ~1 × 10^−9^ Torr and the operating pressure ~1 × 10^−8^ Torr during analysis. Photoelectrons were produced by a SPECS “XR-50” X-ray source, utilising the Mg-kα X-ray anode operating at an energy of 1253.6 eV. Atomic concentrations for each element were determined from their XPS peak areas and their respective sensitivity factors.

**Figure 6:**
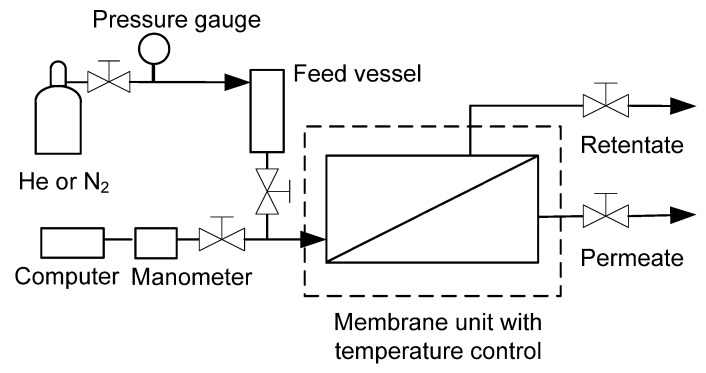
Schematic diagram of the gas permeation system.

Gas permeation (Figure 6) was used to evaluate the intactness of the synthesised zeolite membrane. The membrane was first installed into the stainless steel membrane housing and placed into the unit with temperature control. Permeation of either He or N_2_ was carried out by feeding the gas at 100 kPa to the film-side of the membrane. Pressure decay occurred during the permeation test and was monitored by a TPI 665 digital manometer (Test Products International, Inc. USA). The pressure was recorded by a computer with TPI 665 Data Logger software. Permeation was calculated by normalising the data to the membrane area and the pressure drop measured by the manometer. 

### 3.3. Desalination Test

**Figure 7:**
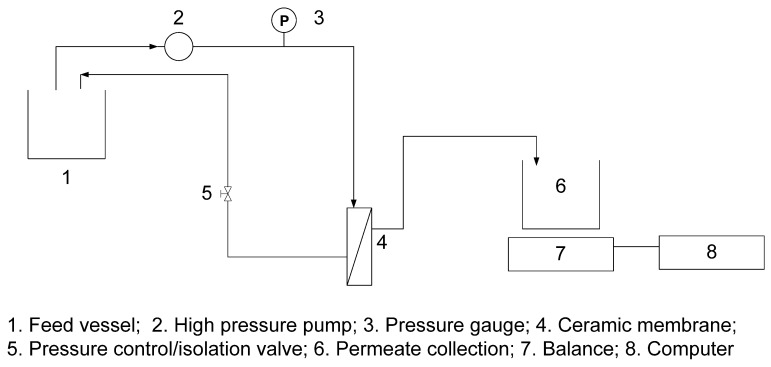
Schematic diagram of the experimental system for membrane desalination.

The desalination performance of the zeolite membrane for NaCl solutions at different applied pressures and temperatures was evaluated in a desalination test system, as shown in Figure 7. The membrane was installed into the same membrane housing as used for gas permeation, and the feed solution (DI water or NaCl solution) was fed at a flow rate of 5 mL·min^−1^ by a high pressure piston pump (Series 1, LabAlliance, USA) with an applied gauge pressure of up to 7 MPa. The NaCl solutions (TDS 3000, 35,000, 50,000, 70,000 and 90,000 mg·L^−1^) were prepared from sodium chloride ACS reagent (≥99.0%, Aldrich). Total dissolved solids (TDS) of the NaCl feed solutions and collected permeate samples were determined with a portable conductivity meter (Sension 156, HACH) and by converting the electrical conductivity measurements to TDS values via a pre-determined relationship. The rejection of Na^+^ was estimated from the TDS values to determine the desalination performance of the membrane.

## 4. Conclusions

A novel MFI-type zeolite membrane was developed by deposition of commercially available zeolite seeds on α-Al_2_O_3_ substrate followed by secondary hydrothermal growth. Desalination through the prepared MFI-type zeolite membrane was investigated for NaCl solutions with different TDS (3000, 35,000, 50,000, 70,000 and 90,000 mg·L^−1^). Good rejection of Na^+^ (~82%) was achieved for an NaCl feed solution (TDS 3000 mg·L^−1^) at an operating pressure of 7 MPa and room temperature by the zeolite membrane. Increasing operating temperature increased the permeation flux of the zeolite membrane, but decreased ion rejection. When increasing the salinity of the feed solution, both Na^+^ rejection and flux were decreased. The flux variations at different salt concentrations can be explained by changes in the effective driving pressure, as the specific flux was constant. SEM measurements confirmed the formation of randomly orientated MFI-type zeolite membrane film on the surface of an α-Al_2_O_3_ support, but showed no changes in structure after desalination testing with NaCl feed solutions. However, XPS elemental analysis detected Na and Cl signals on the surface of the desalination tested membrane, suggesting that interaction between zeolites and ions present in NaCl feed solution might have occurred during desalination. The results obtained in this study showed that MFI-type zeolite membranes have some potential applications, where high rejections (>99.5%), like polymers, are not needed (e.g., brackish water treatment, industrial waste treatment or seawater pre-treatment) and for high temperature desalination. 
